# The impact of a new exercise facility on physical activity at the community level: a non-randomized panel study in Japan

**DOI:** 10.1186/s12889-019-7146-x

**Published:** 2019-06-18

**Authors:** Akio Kubota, Munehiro Matsushita, Ben J. Smith, Takemi Sugiyama, Takashi Arao

**Affiliations:** 1 School of Physical Education, Tokai University, 4-1-1 Kitakaname, Hiratsuka-shi, Kanagawa 259-1292 Japan; 2 Mary MacKillop Institute for Health Research, Australian Catholic University, 215 Spring St, Melbourne, VIC 3000 Australia; 3 School of Public Health and Preventive Medicine, Monash University, 553 St Kilda Road, Melbourne, VIC 3004 Australia; 4 Sydney School of Public Health, University of Sydney, Edward Ford Building (A27) Fisher Road, Sydney, NSW 2006 Australia; 5 Centre for Urban Transitions, Swinburne University of Technology, EW Building, Hawthorn, VIC 3122 Australia; 6 Meiji Yasuda Life Foundation of Health and Welfare, Physical Fitness Research Institute, 150 Tobukimachi, Hachioji-shi, Tokyo, 192-0001 Japan

**Keywords:** Built environment, Sports facility, Community wide intervention

## Abstract

**Background:**

Considering that building a sports facility is a major investment to promote population health, it is important to understand whether it is effective in increasing the level of physical activity (PA) in the community. This study examined the impact of building a new multipurpose exercise facility on community-level PA in Japan.

**Methods:**

This non-randomised panel study compared two sites: an intervention site where a new exercise facility was built (opened after baseline data collection) and a control site where there was no such additional exercise facility. From each site, 3200 adult residents (aged 30–74 years) were randomly selected at baseline (2013) and at follow-up (2015). The number of participants retained for analysis was 845 at baseline and 924 at follow-up for the intervention site, and 821 at baseline and 1018 at follow-up for the control site. The outcomes were participants’ self-reported PA, perceived availability of PA facilities, awareness of others being active, and willingness to engage in PA. We examined the interaction terms between the sites and time of measurement in regression analyses to examine whether the magnitude of change from baseline to follow-up differed between the two sites.

**Results:**

The changes in the proportion of participants meeting the PA guideline and those engaging in moderate-to-vigorous intensity PA were not significantly different between the intervention and control sites. The intervention site had a greater increase in the proportion of participants who were aware of PA facilities from baseline to follow-up than in the control site. The odds ratio for awareness of others being active approached significance, suggesting that there was a tendency at the intervention site towards a greater increase in the proportion of participants who noticed physically active people.

**Conclusions:**

This study did not find community-level increases in PA after the construction of the exercise facility. However, a significant improvement in the awareness of PA facilities was observed in the intervention site. A sustained community-level effort to promote PA, possibly including social components, and a further tracking of residents’ PA are needed to take a full advantage of the new exercise facility and to assess its long-term impact.

**Trial registration:**

UMIN-CTR UMIN000034116 (retrospectively registered: 13 September 2018).

**Electronic supplementary material:**

The online version of this article (10.1186/s12889-019-7146-x) contains supplementary material, which is available to authorized users.

## Background

Regular physical activity (PA) confers a range of health benefits for adults, including reduced risk of developing heart disease, stroke, type 2 diabetes, and some cancers [1]. Despite public health efforts to promote PA, low levels of PA among adults are reported in many countries [2]. In Japan, only 36% of men and 29% of women had a physical activity habit (engaging in at least 30 min of physical activity twice a week for the last 12 months) in 2017, and the prevalence has been stable for the last 10 years [3]. Increasing population-level PA is thus a public health priority. Many PA promotion programs that have been implemented tend to focus on individual motivation to exercise, but such approaches are known to be less successful in sustaining long-term behavioral changes [4]. It is now recognized that strategies focusing only on individuals are unlikely to be sufficient to increase PA at the population level [5]. To promote PA more effectively, the ecological model of health behavior is increasingly being adopted to guide PA interventions [6]. The ecological model posits that factors at multiple levels (individual, social/community, environmental, and policy) influence individual behaviors and that interventions addressing multi-level influences are more effective [7]. A Cochrane review on community-wide interventions to increase PA also supports the importance of environmental components [8]. An important principle of the ecological model is to make participation in PA easy and accessible through various means, such as providing social support, removing barriers, and creating more opportunities to be active.

Exercise and recreational facilities where near-by residents can engage in a range of physical activities can be an important community resource to promote PA. A review of recreational environments and PA found that outdoor recreational facilities such as parks and trails are associated with residents’ PA, but most studies were cross-sectional in design [9]. For instance, better access to sports facilities was found associated with a greater amount of physical activity in Korean adults [10]. A Danish study using GPS and accelerometer also found that having sports facilities nearby (within 800 m from home) was associated with a longer duration of moderate-to-vigorous PA [11]. There are some natural experimental studies examining the impact of improving PA facilities. An Australian study examining the impact of playground refurbishment found increased visitors and active users after restoration [12]. Similarly, a study on park renovation in the U.S. found increased visitation and more energy expenditure by park visitors after park improvement [13]. An observational cohort study in Finland has shown that participants moving to areas where access to sports facilities was poorer (increased distance to and decreased number of them) decreased their PA levels in comparison to before the move [14]. However, a recent systematic review of natural experiments has reported that research has not yet examined the impact of a new exercise facility on residents’ PA [15]. Considering that building a sports facility is a major investment to promote community health, it is important to understand whether it is effective in increasing the level of PA in the community. This study examined the impact of a newly constructed multipurpose exercise facility on community-level PA, perceived availability of PA facilities, awareness of others being active, and willingness to engage in PA in Japan. A focus of this study was on mid-to-older aged adults, who can particularly benefit from additional PA, given that their daily activity levels are lower compared to younger adults [16].

## Methods

### Study design and settings

This study was a non-randomized panel study, in which repeated cross-sectional data were collected from different population samples at baseline and follow-up in an intervention and a control site. The intervention site was Nagaizumi, Shizuoka Prefecture (Population: 41,912; percentage of population aged ≥65 years: 19%, as of April 2013). The control site was Oiso in an adjacent prefecture (Population: 32,625; percentage of population aged ≥65 years: 29%, as of April 2013). The control site was chosen to obtain data from a locality where the population size, geography, and climate were comparable to those of the intervention site and where no major exercise facility development or PA promotion activities were expected to take place during the study period. Since the road distance between the two sites is over 50 km and they are in different prefectures, people in the control site were unlikely to be affected by activities in the intervention site.

### Study protocol

**Fig. 1:**
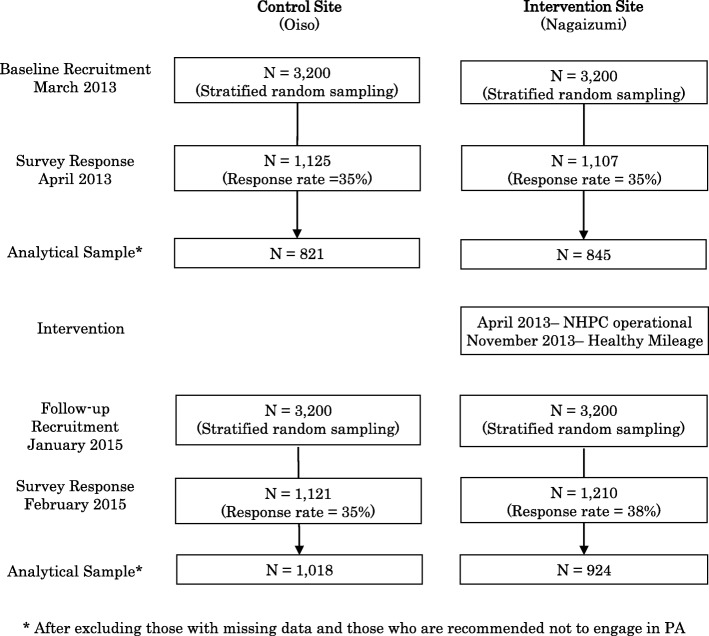
The process of participants’ recruitment and data collection

Complete details of the trial protocol based on the Transparent Reporting of Evaluation with Non-randomized Designs statements have been published elsewhere [17]. In March 2013, 3200 adult residents (aged 30–74 years) were randomly selected in each site from the registry of residential addresses. Individuals were chosen from lists of residents that were classified by age (30–39, 40–49, 50–59, and 60–74 years) and gender. This age group (30–74 years) was chosen as they are known to have a greater risk of developing chronic diseases [18]. Individuals who were selected received a postal invitation to take part in this study and those who agreed to participate received a survey questionnaire. There was no incentive to participate in this study. The baseline survey was returned in April 2013 by 1107 participants in the intervention site (response rate = 35%) and by 1125 participants in the control site (response rate = 35%). The intervention (detailed below) began in August 2013. In January 2015, participants were randomly recruited again. The follow-up survey was returned in February 2015 by 1210 participants in the intervention site (response rate = 38%) and by 1121 participants in the control site (response rate = 35%). Figure 1 illustrates the process of participants’ recruitment and data collection.

### Intervention

The intervention aimed to promote PA for all residents living in the intervention site. The primary intervention was the construction of a new multipurpose exercise facility, the Nagaizumi Health Promotion Center (NHPC). The NHPC, which opened in August 2013, includes indoor facilities (25 m pool, 170 m walking trail, multi-purpose gym, and group exercise rooms) and outdoor facilities (multi-purpose athletic field, 875 m walking trail, and park). All residents of Nagaizumi could use facilities in the NHPC for a small fee (e.g., the use of multi-purpose gym: US$1).

Two months after the opening of the NHPC, the health promotion division of Nagaizumi launched a “Healthy Mileage” reward program. This incentive program, originally developed by the prefectural health promotion office, sought to encourage people to engage in healthy behaviors including PA. All residents of the town were invited to take part in the program. Entrants self-reported whether they met various goals specified in the program, such as “did exercise/sports”, “used stairs rather than an elevator”, and “walked/cycled instead of driving”. A point (an official stamp) was given to each goal achieved at town facilities including the NHPC. The points that residents earned through this scheme could be used to pay for entrance to the NHPC or to receive discounts at local grocery stores.

No special PA promotion program was implemented in the control (Oiso) site during the study period. The NHPC is not easily accessible from Oiso. However, the health promotion division of Oiso provided routine promotion activities including health education classes and health promotion events.

### Measures

The primary outcome was participants’ self-reported PA. It was measured at baseline and follow-up using the Japanese version of the International Physical Activity Questionnaire Short-Form (IPAQ). The IPAQ was a self-administered questionnaire in which participants were asked to report time spent engaging in various intensities of PA over the preceding 7 days. The Japanese version has been shown to have acceptable validity and reliability [19]. Total weekly energy expenditure expressed in metabolic equivalent (MET·hour/week) was calculated following the IPAQ protocol (including truncation of excessive physical activity). Since the distribution of energy expenditure was highly skewed, categorical measures were used as PA outcomes. One PA outcome was whether or not participants met the Japanese PA guideline (total energy expenditure ≥23 MET·hour/week) [20]. The other was whether participants engaged in moderate-to-vigorous intensity physical activity (MVPA, not including walking). This measure allowed us to examine whether the NHPC contributed to reducing the number of people who are totally inactive.

The secondary outcomes were perceptual and motivational factors. Perceived availability of PA facilities, awareness of others being active, and willingness to engage in PA were assessed using the following items: 1) My neighborhood has several free or low-cost recreation facilities, such as parks, walking trails, bike paths, recreation centers, playgrounds, and public swimming pools; 2) I see many people being physically active in my neighborhood; and 3) How willing are you to engage in PA? The response format ranged from 1 (strongly disagree) to 4 (strongly agree) for the first two items, and from 1 (not at all) to 4 (much) for the third item. Participant’s response for each item was dichotomized into 0 (response 1 and 2) and 1 (response 3 and 4), with the latter indicating availability of PA facilities, being aware of others engaging in PA, and being willing to engage in PA.

The demographic information collected from participants included gender, age, education level (12+ years of education or less), marital status (single or couple), employment status (working or not), body mass index (BMI) calculated from self-reported weight and height (kg/m^2^), and self-rated health status (4-point scale ranging from bad to good). Participants also self-reported their overall health status on a 4-point scale, in response to the question “Overall, how would you rate your health?”. The response was dichotomized into bad health (scores 1 and 2) and good health (scores 3 and 4). In addition, participants were asked whether they were told by a doctor not to engage in PA to exclude those who were unable to conduct any PA. In addition, participants were asked whether they were told by a doctor not to engage in PA to exclude those who were unable to conduct any PA.

### Statistical analyses

To evaluate the effectiveness of the intervention, the changes in the outcomes from baseline to follow-up were compared between the intervention and control sites, following previous studies with the same panel design [21, 22]. The outcomes were compared using chi-square test for unadjusted analyses and logistic regression for adjusted analyses. To examine whether the magnitude of change differed between the intervention and control sites, we used the interaction term between the sites and the time of measurement (baseline or follow-up). The coefficient for the interaction term indicated to what extent the intervention site “improved” in each outcome from baseline to follow-up relative to the control site [22]. These models adjusted for baseline gender, age, education level, marital status, employment status, BMI, and self-rated health. In addition, we conducted analyses stratified by gender and by age (30–59 years and 60–74 years) to check whether the intervention worked differently for sub-groups. Analyses were conducted using IBM SPSS version 24 (IBM, Armonk, NY). Statistical significance was set at *p* <  0.05.

## Results

Table 1 shows the baseline and follow-up characteristics of participants in the intervention and control sites. After excluding participants who were told by a doctor not to engage in PA and those who had missing data in the outcomes or demographic variables, the number of participants retained for analysis was 821 for the intervention site and 845 for the control site at baseline, and 1018 for the intervention site and 924 for the control site at follow-up. The number of participants at baseline was smaller than that at follow-up. This was mainly because the baseline survey was unclear about how to answer the gender question. This problem was rectified in the follow-up survey. At baseline, participants in the intervention site were more likely to have lower educational attainment and to be working, compared to those in the control site. At follow-up, participants in the intervention site were more likely to be younger, to have lower educational attainment, to live alone, and to be working, compared to those in the control site.

**Table 1:** Characteristics of participants in the intervention and control sites at baseline and follow-up

Items	Baseline		P	Follow-up		P
	Intervention site	Control site		Intervention site	Control site	
N	821	845	–	1018	924	–
Age, Mean ± SD	52.9 ± 12.5	53.1 ± 12.5	0.68	52.1 ± 12.3	53.2 ± 12.4	0.04
Gender, % Men	40.9	42.0	0.65	43.3	46.0	0.24
Education level, % > 12 years	51.8	65.1	< 0.001	55.8	67.6	< 0.001
Marital status, % Single	7.6	5.9	0.18	9.1	6.1	0.01
Employment status, % Working	65.4	58.9	0.01	69.7	65.2	0.03
BMI, Mean ± SD	22.3 ± 3.3	22.2 ± 3.1	0.64	22.2 ± 3.2	22.3 ± 3.1	0.68
Self-rated health, % Good ^a^	88.6	89.6	0.50	87.7	88.4	0.64

^a^ Scores 3 and 4 on a scale ranging from 1 (bad health) to 4 (good health)

During the study period, the NHPC was used by 275 persons/day in 2013 and 443 person/day in 2014. The NHPC organized two major promotion events per year, attracting in total 1100 persons in 2013 and 600 persons in 2014 (in addition to daily users of the NHPC). The Healthy Mileage program usage was 311 times in 2013 (less than once per day) and 986 times in 2014 (2.7 times/day).

Table 2 shows the change in each outcome measure from baseline to follow-up for the intervention and control sites, and the adjusted differences between the sites at baseline and follow-up. Odds ratios greater than 1 indicate that the corresponding figure is larger in the intervention site than in the control site. The proportion of participants meeting the PA guideline decreased slightly from baseline to follow-up both in the intervention site and the control site. The same was true for the percentage of people who engaged in MVPA. This may be because the follow-up survey was conducted in February, which was slightly colder than April when the baseline survey was conducted. The intervention site had a marginally lower proportion of participants meeting the PA guideline than the control site at baseline, but it was not significantly different at follow-up. The two sites did not differ in the percentage of those who engaged in MVPA at baseline or follow-up. The proportion of those who knew of PA facilities nearby was significantly higher in the intervention site than the control site both at baseline and follow-up. The proportion of those who were aware of others being active was significantly lower in the intervention site than the control site at baseline, but it was not significantly different at follow-up. The intervention and control sites did not differ in participants’ willingness to engage in PA both at baseline and at follow-up.

**Table 2:** The adjusted differences between the intervention and control sites at baseline and follow-up

Items	Intervention site			Control site			Adjusted differences between the intervention and control sites: Odds ratios (95%CI) ^b^	
	Baseline *n* = 821	Follow-up *n* = 1018	Change ^a^	Baseline *n* = 845	Follow-up *n* = 924	Change ^a^	Baseline	Follow-up
% of those who met the PA guideline	20.2	18.5	−1.7	23.2	21.1	−2.1	0.80 (0.63, 1.01)†	0.83 (0.66, 1.04)
% of those who engaged in MVPA	42.6	39.6	−3.0	44.5	43.3	−1.2	0.95 (0.78, 1.16)	0.88 (0.73, 1.05)
% of those who perceived availability of PA facilities	76.4	81.2	4.8	68.2	67.0	−1.2	1.59 (1.28, 1.99)**	2.34 (1.89, 2.90)**
% of those who were aware of others being active	85.7	87.8	2.1	89.8	88.4	−1.4	0.71 (0.52, 0.96)*	1.02 (0.77, 1.35)
% of those who were willing to engage in PA	87.0	85.0	−2.0	87.5	86.6	−0.9	1.04 (0.77, 1.39)	0.94 (0.72, 1.22)

† *p* < 0.1, * *p* < 0.05, ** *p* < 0.01

^a^ Follow-up minus baseline

^b^ Analyses adjusted for gender, age, education level, marital status, employment status, BMI, and self-rated health (reference: control site)

Table 3 shows the regression coefficients of the interaction terms between the sites and the time of measurement, which can be interpreted as the adjusted differences in the change (from baseline to follow-up) between the intervention and control sites. Odds ratios greater than 1 denote that the intervention site had a greater improvement in the outcome of interest than did the control site. The changes in the proportion of participants meeting the PA guideline and those engaging in MVPA were not significantly different between the intervention and control sites. The intervention site had a greater increase in the proportion of participants who were aware of PA facilities from baseline to follow-up than in the control site. The odds ratio for participants’ awareness of others being active approached significance (*p* = 0.09), suggesting there was a tendency at the intervention site towards a greater increase in the proportion of participants who noticed physically active people from baseline to follow-up. There was no significant difference between the intervention and control sites in terms of the changes in participants’ willingness to engage in PA. The results of analyses stratified by gender and by age (30–59 years and 60–74 years) are shown (see Additional file 1). Gender-specific analyses found that more women in the intervention site became aware of PA facilities at follow-up than did those in the control site. No significant differences were observed for men. Age-specific analyses found a greater increase in the proportion of older participants (60–74 years) who were aware of PA facilities and aware of others being active from baseline to follow-up in the intervention site, relative to the control site. No significant differences were observed for younger adults (30–59 years).

**Table 3:** Differences in outcome changes from baseline to follow-up between the intervention and control sites

Items	Odds ratios for the interaction term between the site and time ^a^	P
% of those who met the PA guideline	1.01 (0.86, 1.19)	0.91
% of those who engaged in MVPA	0.96 (0.84, 1.09)	0.51
% of those who perceived availability of PA facilities	1.20 (1.03, 1.40)	0.02
% of those who were aware of others being active	1.19 (0.97, 1.46)	0.09
% of those who were willing to engage in PA	0.96 (0.79, 1.17)	0.69

^a^ Analyses adjusted for gender, age, education level, marital status, employment status, BMI, and self-rated health (reference: control site)

## Discussion

This study assessed the impact of building a new multipurpose exercise facility on a community-level PA. Although there have been some natural experimental studies investigating the effect of park improvements [12, 13], no research to date appears to have examined whether building a new exercise facility increases PA at the community level [15]. During the two-year study period, the intervention site, where the new exercise facility was built, did not show a significant increase in the PA measures relative to the control site. Similar non-significant changes in PA have been observed in other studies involving community-wide interventions [21, 23–25]. A review of community-based PA interventions also reports inconsistent findings with regard to their effects in increasing PA [8].

A potential reason for not finding significant increases in PA measures, despite a relatively large number of NHPC users, is that the new exercise facility may have attracted people who were already active elsewhere, rather than encouraging non-active people to initiate PA. The ecological model holds that multi-level interventions (involving individual, social, and environmental influence) are more effective, than single-level interventions [6]. A natural experiment examining park renovation found that modifications to the physical improvement alone are not enough to increase PA; programs that facilitate people to be active are also needed [26, 27]. Although the intervention examined in this study was multi-level, involving individual (incentive by the Healthy Mileage program) and environmental (the NHPC) factors, the uptake of the Healthy Mileage program was very low. A stronger incentive program that would reach a broader population may have been needed to take advantage of the new exercise facility. A social element was also lacking in the intervention implemented in this study. Additional strategies, such as involving existing community groups, working with community champions, and the use of social media, may have improved the reach and effectiveness of the intervention.

The study found that more people in the intervention site became aware of PA facilities at follow-up, compared with those in the control site, which was potentially due to media coverage of the NHPC. Gender- and age-specific analyses found that this was particularly the case with women and older adults. It can be argued that more women and older adults became aware of the facilities as they possibly spent longer time in their neighborhoods and were more exposed to local information. Given that women and older adults tend to be less active compared to their counterparts [28], the increased awareness of PA opportunities can be used as a foundation for increasing PA among them in future intervention efforts.

The study also found a marginally significant increase in the proportion of people who were aware of others being active in the intervention site. However, research appears to be mixed in the role of recognizing other people being active as a correlate of PA [29–31]. Considering that participants’ willingness to engage in PA did not improve in the intervention site, the construction of the new exercise facility may not have been strong enough to change residents’ attitudes towards participating in PA. It can be also argued that a two-year study period was not long enough to change residents’ motivational factors relevant to PA. The review on community-based PA interventions mentioned above reported that the median intervention period of the studies identified was 3 years (range: 1 to 7 years) [8]. Continuing promotion efforts (and long-term observation) may be needed to achieve a shift in the stages of PA [32].

A strength of this study was that we collected PA data before and after the construction of a new exercise facility, with comparable PA data from the control site where no special PA promotion was conducted during the study period. This study used a panel design, where data were collected at baseline and follow-up, rather than conducting a cohort study. This methodological decision was made to assess PA participation at the community level consistently at baseline and follow-up. Although participants recruited at baseline and at follow-up were somewhat different in socio-demographic characteristics (Table 1), random sampling at both time points can reduce selection bias. This is potentially an advantage of a panel design in contrast to a cohort design, which could introduce systematic attrition bias at follow-up. A study limitation was the use of self-reported PA measures, which may be subject to recall error and desirability bias. An objective PA measure using activity monitors is needed for future research to produce more robust evidence. Another issue was that the baseline and follow-up data were collected in different months (April for baseline, February for follow-up) due to a funding requirement to end the project before March 2015. However, we do not think this is a major limitation as the study compared the intervention and control sites in their changes in physical activity (not simply the magnitude of change from baseline to follow-up). The study did not consider the effect of existing facilities. Although the NHPC was the first multipurpose exercise facility in Nagaizumi, it is unknown whether the existing recreational facilities were meeting the residents’ demands for physical activity. It is also unknown whether new exercise facilities opened in adjacent localities or existing facilities were renovated during the study period, which may have influenced the results. The study was conducted in a regional city with a small population. The introduction of a new exercise facility in an urban context may produce different effects. We were unable to assess the impact of distance to the exercise facility. Future research can investigate the distance to a facility to understand the size of a “catchment” area, within which residents are more likely to use the facility.

## Conclusions

This study found that the construction of a new exercise facility in a regional city in Japan did not generate an increase in community-level PA during the study period. However, significant improvement in awareness of recreational facilities was observed in the intervention site, suggesting that the new facility may have raised awareness of PA opportunities, which could over a period help some people to initiate PA. A sustained community-level effort to promote PA, possibly including social components, and a further tracking of residents’ PA are needed to take advantage of the new exercise facility and to assess its long-term impact.

## Additional file

Additional file 1: **Table S1.** Differences in outcome changes from baseline to follow-up between the sites (men). **Table S2.** Differences in outcome changes from baseline to follow-up between the sites (women). **Table S3.** Differences in outcome changes from baseline to follow-up between the sites (aged 30–59 years). **Table S4.** Differences in outcome changes from baseline to follow-up between the sites (aged 60–74 years). (DOCX 20 kb)

## Data Availability

The datasets used and/or analyzed for the current study are not publicly available due to data sharing not being approved by Nagaizumi and Oiso local governments, but are available from the corresponding author if approved by these local governments.

## Abbreviations

MVPA: Moderate-to-vigorous intensity physical activity
NHPC: Nagaizumi Health Promotion Center
PA: Physical activity
