# Supplementary material for: The impact of a new exercise facility on physical activity at the community level: a non-randomized panel study in Japan
Source: BMC Public Health. 2019 Jun 18;19:777. doi: 10.1186/s12889-019-7146-x (PMC6582471; doi:10.1186/s12889-019-7146-x)
Supplement: Supplementary file 1 — Table S1. Differences in outcome changes from baseline to follow-up between the sites (men). Table S2. Differences in outcome changes from baseline to follow-up between the sites (women). Table S3. Differences in outcome changes from baseline to follow-up between the sites (aged 30–59 years). Table S4. Differences in outcome changes from baseline to follow-up between the sites (aged 60–74 years). (DOCX 20 kb) [file 12889_2019_7146_MOESM1_ESM.docx]

| **Table S1** Differences in outcome changes from baseline to follow-up between the sites (men) | | |  |
| --- | --- | --- | --- |
| Items | Odds ratios for the interaction term  between the site and time ^a^ | P values | |
| % of those who met the PA guideline | 1.05 (0.83, 1.32) | 0.69 | |
| % of those who engaged in MVPA | 0.98 (0.80, 1.20) | 0.83 | |
| % of those who perceived availability of PA facilities | 1.01 (0.80, 1.28) | 0.93 | |
| % of those who were aware of others being active | 1.10 (0.81, 1.51) | 0.54 | |
| % of those who were willing to engage in PA | 0.79 (0.58, 1.07) | 0.13 | |
| ^a^ Analyses adjusted for age, education level, marital status, employment status, BMI, and self-rated health (reference: control site) | | | |

| **Table S2** Differences in outcome changes from baseline to follow-up between the sites (women) | | |
| --- | --- | --- |
| Items | Odds ratios for the interaction term between the site and time ^a^ | P values |
| % of those who met the PA guideline | 0.97 (0.77, 1.22) | 0.79 |
| % of those who engaged in MVPA | 0.94 (0.79, 1.13) | 0.52 |
| % of those who perceived availability of PA facilities | 1.34 (1.10, 1.64) | 0.004 |
| % of those who were aware of others being active | 1.26 (0.96, 1.65) | 0.10 |
| % of those who were willing to engage in PA | 1.12 (0.87, 1.45) | 0.37 |
| ^a^ Analyses adjusted for age, education level, marital status, employment status, BMI, and self-rated health (reference: control site) | | |

| **Table S3** Differences in outcome changes from baseline to follow-up between the sites (aged 30–59 years) | | |
| --- | --- | --- |
| Items | Odds ratios for the interaction term between the site and time ^a^ | P values |
| % of those who met the PA guideline | 1.02 (0.83, 1.26) | 0.82 |
| % of those who engaged in MVPA | 0.99 (0.84, 1.17) | 0.89 |
| % of those who perceived availability of PA facilities | 1.11 (0.93, 1.33) | 0.26 |
| % of those who were aware of others being active | 1.07 (0.84, 1.36) | 0.58 |
| % of those who were willing to engage in PA | 0.94 (0.75, 1.19) | 0.62 |
| ^a^ Analyses adjusted for gender, education level, marital status, employment status, BMI, and self-rated health (reference: control site) | | |

| **Table S4** Differences in outcome changes from baseline to follow-up between the sites (aged 60–74 years) | | |
| --- | --- | --- |
| Items | Odds ratios for the interaction term between the site and time ^a^ | P values |
| % of those who met the PA guideline | 1.02 (0.78, 1.34) | 0.89 |
| % of those who engaged in MVPA | 0.90 (0.71, 1.13) | 0.36 |
| % of those who perceived availability of PA facilities | 1.44 (1.09, 1.90) | 0.009 |
| % of those who were aware of others being active | 1.62 (1.09, 2.42) | 0.02 |
| % of those who were willing to engage in PA | 1.03 (0.71, 1.47) | 0.89 |
| ^a^ Analyses adjusted for gender, education level, marital status, employment status, BMI, and self-rated health (reference: control site) | | |
